# Book Reviews

**Published:** 1893-02

**Authors:** 


### *BOOK REVIEWS.*

---

Anatomy (Double Number). By Fred J. Brockway, M. D., Assistant Demonstrator of Anatomy, College of Physicians and Surgeons, New York, and A. O'Malley, M. D., Instructor in Surgery, New York Polyclinic. Being Volume I. of the Students' Quiz Series, edited by Bern B. Gallaudet, M. D., Demonstrator of Anatomy, College of Physicians and Surgeons, New York; Visiting Surgeon, Bellevue Hospital, New York. Pocket-size 12mo., 367 pages, 15 illustrations. Limp cloth, $1.75. Philadelphia, Lea Brothers & Co.

We have always questioned the utility of quiz compends, and certainly some of them that have been prepared have served no good purpose. The present volume, though designed for students, can hardly be called a quiz compend. It is the whole subject of human anatomy carefully and judiciously condensed. It is no easy matter to prepare such a book, and we think the authors have succeeded as well as the difficulties of the undertaking would permit. It is the best abridgment of the subject which we have ever seen. The compilers have followed pretty closely the works of Quain, Gray and Henle, the order and classification of the latter being adopted. In places there is unnecessary mention of unimportant details, such as rare departures from the usual arrangement of parts, rudimentary muscles, etc., and occasionally a typographical error. We think it is time for us to abandon the descendens *noni* nerve ; descendens *hypoglossi* is certainly preferable. A useful glossary is found at the close of the volume. The index should be more complete.

---

International Clinics. A quarterly of clinical lectures. Edited by John M. Keating, M. D., Colorado Springs; Judson Daland, M. D., Philadelphia; J. Mitchell Bruce, M. D., London; and David Finlay, M. D., Aberdeen. Volumes I, II and III. J. B. Lippincott Company, Philadelphia.

This excellent series of clinical lectures has been reviewed in these pages often enough not to require very extended notice now. The three volumes before us belong to the series of 1892, and are full of useful and practical lectures for the practitioner. The advantage of these clinics for the physician lies largely in the fact that they are so entirely practical. Actual cases are reported in an entertaining style, and the treatment indicated in particular cases is carefully described. Wherever it has been thought advisable photographs have been inserted, and these add much to the value of the "Clinics." The lectures are all on subjects of great practical interest to the physician, and must occupy a high rank in the current medical literature of the day. We are pleased to note the continued success of this publication. In this series the name of Dr. Daland has been substituted for that of Dr. J. P. Crozier Griffith.

---

ANNUAL OF THE UNIVERSAL MEDICAL SCIENCES. A yearly report or the progress of the general sanitary sciences throughout the world. Edited by Charles E. Sajous, M. D., and seventy associate editors. Five volumes. 1892. F. A. Davis Company, 1231 Filbert St., Philadelphia.

*The Annual of the Universal Medical Sciences* has been reviewed every year in these pages, and has come to be so well known to the profession that we need not say very much about it here. This is the fifth series of its publication. With the same degree of excellence which characterized its predecessors this series carries out the original plan and scope of the work in furnishing a condensed and useful *résumé* of the progress made within the year in all the departments of medical and sanitary science. Dr. Sajous and his able staff of assistants have labored diligently over their work, and its continued success demonstrates that it is being appreciated.

---

A MANUAL OF THE PRACTICE OF MEDICINE. Prepared especially for students, by A. A. Stevens, A. M., M. D., Instructor of Physical Diagnosis in the University of Pennsylvania. Illustrated. 500 pages. W. B. Saunders, 913 Walnut St., Philadelphia.

A manual cannot begin to do justice to the broad subject of the practice of medicine. This work is about on a par with others of its class, and is a very condensed outline of practice. The author admits to have drawn freely from a long list of authorities, and we judge that he has. The section on diseases of the skin, occupying a large space, is decidedly out of place. Students may find it of some service in preparing for examination. Beyond that it will serve no good purpose. We must confess that the function and force of the poetical selection on the title page we cannot appreciate.

